# Dentists’ perceptions and usability testing toward the implementation of the ISAC, a comprehensive oral cancer intervention in dental practices: a qualitative study in Jazan region, Saudi Arabia

**DOI:** 10.1186/s12913-022-07586-2

**Published:** 2022-02-12

**Authors:** Ibtisam Moafa, Ciska Hoving, Bart van den Borne, Mohammed Jafer

**Affiliations:** 1 Dental Public Health Division, Department of Preventive Dental Sciences, College of Dentistry, Jazan University, Jazan, Saudi Arabia; 2 Department of Health Promotion, Maastricht University/CAPHRI, PO Box 616, 6200 MD Maastricht, the Netherlands

**Keywords:** Early detection of cancer, Oral cancer, Behavior change, Public health dentistry, Self-examination, Think aloud

## Abstract

**Objective:**

We aimed to explore dentists’ perceptions toward the implementation of a comprehensive intervention (ISAC) for the early detection and prevention of oral cancer in a dental clinic.

**Methods:**

The ISAC intervention was presented to ten purposefully sampled dentists in Jazan Dental School (JDS). Participating dental interns were asked to practice the ISAC intervention whilst thinking aloud. A semi-structured interview technique was used to allow free expression of participants’ perceptions related to the ISAC intervention and to control the flow of topics. Fleuren’s framework theory informed the analysis. The interviews were transcribed verbatim and analyzed using the deductive-inductive framework analysis.

**Results:**

Practicing the ISAC intervention was perceived to enhance the early detection and prevention of oral cancer. Serving community needs and engaging community groups were perceived to be related to a high relevance and compatibility of the ISAC intervention. Being a comprehensive intervention with well-defined objectives and being built on relevant data from the participants’ community and having dentists as a target group were the perceived relative advantages of the ISAC intervention compared to other programs. A supportive environment, gender-concordance, use of regional trainers, standard examination form and collaboration with other sectors were perceived to be the facilitators. Competition with clinical time, use of different examination forms and low organizational leader interests were perceived as impeding factors against effective implementation in a real-world context. Reward, easy to practice, feeling confidence and satisfaction, advertisement as well as use of a role model approach were perceived to be motivating factors.

**Conclusions:**

Integrating data from representers of different participant groups during intervention conceptualization and development are critical for the intervention compatibility and acceptability. The study findings showed the opportunities of intertwining the intrinsic motivators of satisfaction and altruism existing in the target group and the extrinsic motivator of official diagnostic skill, certification that may boost and sustain the behavior change. Intervention features that influence perceived relevance, compatibility, relative advantage and motivation may be of great importance for intervention practice.

**Supplementary Information:**

The online version contains supplementary material available at 10.1186/s12913-022-07586-2.

## Background

The global trend of oral cancer (OC) changes overtime. OC is the sixth common malignancy worldwide with 274,300 annual new cases [1]. OC has its highest burden on the Asian continent in comparison to the other continents due to distinguishing cultural practices in Asian countries like chewing tobacco, extensive betel quid use in combination with alcohol consumption, which are considered main risk factors of OC [2]. OC is highly prevalent in developing countries especially south Asian countries encompassing Pakistan, India and Bangladesh [3]. Among the gulf countries, the southern region of Saudi Arabia -Jazan- has the highest cases of OC where a special type of smokeless tobacco (Shammah) is commonly used among the Jazan population [4]. OC impact on the population is assessed through the incidence rate, mortality rate or survival rate [3]. Due to the current advances in technologies, OC is diagnosed at an earlier stage of the disease [3].

Several studies revealed that the early detection of OC leads to better prognosis of the disease and better survival rates [5–7]. Previous studies among dental faculty members, dental students and dental interns revealed an existing gap between knowledge and actual practice in OC screening. Dentists and dental interns failed to perform OC screening although they had adequate knowledge and a favorable perception toward OC screening [8, 9]. In a recent study we showed a statistically significant difference in diagnosis of OC in favor of dental faculty members who received specialized training [8]. Lack of confidence in ability to detect premalignant lesions and on providing tailored patient education about OC risk factors was the common factor perceived by participants to be related to their observed passive behavior [8, 10, 11]. Moreover, we found that dental interns had the lowest score in relation to OC screening [8].

Pre-implementation evaluation of a developed health intervention is critical for the ultimate success of an intervention [12]. Tailoring interventions to the target group characteristics, to the intervention special context and to the target health professionals is highly recommended by previous research [12–14]. However, this is rarely performed as most healthcare interventions were solution-driven and not needs-driven [12]. Even when a pre-implementation study was done, the outputs were frequently not integrated in the intervention. Health interventions often need to be adapted to fit the local context for implementation success [13]. Intervention adoption and implementation are influenced by several factors. These factors involve characteristics of the intervention, characteristics of the adopter as well as contextual factors [15, 16]. Adopters are the individuals but also can be organizations or hospitals who decide to commit to or to initiate the evidence-based intervention [14]. While implementers are those individuals who are responsible of delivering the intervention to the target group [14]. The adopters’ awareness and attitude toward the intervention can influence its uptake and effective use [13]. Similarly, the implementers’ knowledge, skills and attitudes toward the evidence-based intervention influence the ability to implement the intervention with fidelity [13, 14]. Fidelity measures the extent to which the evidence-based intervention is implemented according to the protocol [13]. When the implementer lacks the knowledge, skills or confidence, the amount and the quality of the delivered intervention will be compromised [13, 14]. Alternatively, highly experienced implementers might have high confidence in their abilities and tend to change the intervention elements and do not adhere to the intervention protocol [13, 14]. Thus, implementers and adopters’ characteristics is vital for the implementation fidelity and eventually the success of intervention. Implementing healthcare interventions is challenging and therefore require careful planning, inclusion of adopter, implementer and target group perceptions, expectations and perceived challenges of the intervention [15].

As part of the pre-implementation phase of a project to increase the early detection and prevention of OC, this paper explored dentists’ perceptions toward the implementation of a comprehensive intervention (ISAC) in their daily practice in dental clinics. The present study answered two research questions: 1-What are the first thoughts, feelings or impressions of dental interns while practicing the ISAC intervention? And 2-What are the adopters, implementers and dental interns’ perceptions toward the ISAC intervention?

## Methods

### Study design, participants and setting

We followed in this qualitative cross-sectional study we followed the Consolidated Criteria for Reporting Qualitative Research Checklist (COREQ) [17]. For the present study we included participants from dental interns, clinical director, intern supervisor as well as staff from community Dentistry Division (CDD). All participants were invited to participate through an internal channel (e-mails). The adopters and implementers were purposively invited to participate in the study as they were the qualified individuals in the Jazan Dental School (JDS) who can decide to adopt and implement the intervention. We selected the JDS setting because it is the major dental institution in Jazan region that provides dental services in different specialties with approximately 80 annual graduates. Thousands of patients across different Jazan governorates are receiving their free dental care in JDS. Further, graduates of JDS are now serving most public and private dental practices across the region. Therefore, it is viewed as the most suitable setting in this stage of intervention mapping focusing on pre-testing the evidence-based intervention design and components. We included the four individuals who were responsible for the adoption and implementation of the ISAC in the JDS setting (100% the population included). With regard to the target group, we included six out of 80 dental interns (7.5% of the population included). We believe six interns is adequate at the current stage of the intervention, as it yielded varied enough information to proceed on to the first trial of the ISAC. Furthermore, the hallmark of qualitative research is the particularity rather than generalizability of findings [14].

### The ISAC intervention

The ISAC stands for: I = inform patient of OC screening; S = screen for OC; A = advice and educate patient of OC and quit its associated risk factors; and C = connect high-risk patients and suspicious cases to specialized centers. Guided by the Intervention Mapping Approach (IMA), the ISAC intervention was developed in a systematic and an iterative process by a multidisciplinary team of researchers, stakeholders and representatives from the target group [14]. IMA is a planning model that utilizes theory and evidence-based methods to develop and evaluate health interventions to promote behavioral and environmental health conducive changes [14]. The ISAC rests on relevant evidence from comprehensive needs’ assessments including participants from dental students, dental interns, college dean, academic staff and dental patients [8–11, 18]. In our context, intervention adopters are the clinical director and the intern supervisor as they are the authority in charge of dental interns and dental clinics management. Staff of the CDD are the intervention implementers and dental interns are the target group of the intervention.

The ISAC intervention comprised the following two sessions: a theoretical session and a practical session. The theoretical session covered 1- Introduction to the ISAC, its relevance and its development; 2- General and local OC epidemiology and risk factors; 3- Full OC screening protocol; 4- Importance of patient education practice; 5- Tobacco counseling by dentists; 6- Patient communication skills; and 7- patient connection to necessary services and centers. While the practical session was constructed as the following: 1- Actual modeling of the ISAC by change agents: inform patient of OC examination, screen for OC, educate and provide brief tobacco counseling and connect patient to specialized tobacco cessation/oncology centers if needed; 2- Second group observing first group performing the ISAC; 3- First group observing second group performing the ISAC.

### Procedures

To answer the first research question (What are the first thoughts, feelings or impressions of dental interns while practicing the ISAC?): we utilized the think-aloud approach during which participating dental interns were instructed on how to think aloud during their modeling of the ISAC intervention on patient-actors [19]. Our role as researchers in the thinking-aloud was to record the expression elicited by the dental interns while they were performing the ISAC sub-behaviors and to prompt them if required (if staying silent). Further, to encourage dental interns through positive reinforcement “you are providing relevant information, good job”. The adopters’ role in the think-aloud was to have the chance to experience the full trialability of the intervention that they decided to adopt in their organization and then express their thoughts and perceptions toward its implementation and potential barriers and facilitators. The implementers’ role was to experience the trialability of intervention they committed to implement (deliver) to dental interns and reflect on their thoughts and perceived difficulties toward its implementation in the JDS. After the thinking-aloud, a focus group discussion was conducted among the participating dental interns to express their thoughts and opinions away from the influence of adopters and implementers. Adopters and implementers were not part of the focus group discussion that was conducted among the dental interns to allow dental interns express their opinions comfortably. The fact that a think-aloud method will always exclude some thought processes that are not held long enough to be expressed in working memory, a follow up interview is commonly recommended to add in-depth information of participants thought processes and to allow interviewees to validate researchers’ interpretation of their think-aloud utterances [20]. Individual one-to-one semi structured qualitative interviews were conducted for the adopters and implementers to reflect on their perceptions toward the ISAC intervention (see Additional file 1). The interviews lasted between 30 and 45 min. While the focus group discussion lasted approximately 50 min. The interviews and FGD were meant to answer the second research question: What are the adopters, implementers and dental interns’ perceptions toward the ISAC intervention? The interviews and FGD were audio-recorded and verbatim transcribed into Arabic language and then converted into the English language and reviewed by bilingual speakers.

### Theoretical framework and measures

The semi-structured guide was developed and guided by the Fleuren framework for determinants of innovation within health care organizations [15]. In 2004, Fleuren and her colleagues have identified 50 potentially relevant determinants of implementation based on a Delphi study and literature review [15]. The list of 50 determinants was subsequently reduced to include 29 determinants [21]. In Fleuren’s framework, the potential determinants were classified according to the following categories: 1) the innovation: compatibility, observability, complexity; 2) the potential user: knowledge, attitude, self-efficacy; 3) the organization: available time, resources, staff capacity; and 4) the socio-political context such as the regulation [15, 21].

Therefore, the semi-structured interview guide covered ten themes in regard to the second research question as the following: knowledge, attitude, barriers, facilitators, change beliefs, motivators, the ISAC development process, intervention characteristics, organizational factors and socio-political factors.

### Data analysis

Data was processed using NVivo software version 12. Framework analysis (FA) was the followed principle in the data analysis [22]. We have utilized this analysis because it is suitable particularly for studies that have a defined sample and predetermined themes, as was the case in the present study. FA also enables the emergence of novel themes. Data analysis included familiarizing with the data through listening to the records and re-reading the transcripts by the first author and the last author, identifying recurrent themes and sub-themes in an iterative process with mutual discussion which included checking the consistency of coding as well as developing and refining the thematic framework. Finally, illustrative quotes for each theme were created. In addition, we’ve utilized a member checking strategy to further enhance the validity of the findings [23]. Therefore, we contacted a subsample of the participants to review the results after the initial themes were identified. They were asked to comment if they felt that their views were fully represented and if they agreed with the interpretation of their quotes. Participants stated that they agreed with the authors interpretation.

## Results

### Participants

Ten participants of both genders participated in the study: six dental interns, two adopters and two implementers. Four individual face-to-face interviews and one focus group discussion was conducted after the thinking-aloud procedure. Table 1 describes participant characteristics and provides more information on data collection method.

**Table 1:** Participants’ demographic and data collection method

	Participant	Number	Gender	Age	Data collection
Adopter	Clinical director	1	M^a^	38	Interview
Adopter	Intern Supervisor	1	M	37	Interview
Implementer	Community Dentistry Staff	2	M and F^b^	M: 40 F: 36	Interview
End-user	Dental Interns	6	3 M and 3 F	23–26	Focus group discussion

^a^M = Male, ^b^F=Female

### Themes

The identified themes were as the following: seven themes were identified on adopting person determinants level, four themes were identified on innovation determinants level, three themes were identified on organization determinants level, two themes on socio-political context level and one theme was developed focusing on recommendation and areas for re-adaptation (please insert Table 2 here). The identified themes were explained in line with the Fleuren framework and summarized as the following (please insert Table 3 here for detailed description of themes and illustrative quotes):

**Table 2:** Summary of themes

Research Question	Theme	Description
1- What are the first thoughts, feelings or impressions of dental interns while practicing ISAC?	The immediate feel	First impressions during ISAC practice (sequence of ISAC steps, performance, feelings)
	Recommendations	ISAC clinical protocol to enhance ISAC practice Separate training on tobacco counseling and patient communication in Arabic language
2- What are the adopters, implementers and dental interns’ perceptions toward ISAC intervention?	Knowledge	Adopters and implementers knowledge toward ISAC intervention
	Perceptions	Adopters and implementers beliefs toward ISAC intervention (attitude, change, facilitators and barriers of adoption/implementation)
	ISAC characteristics	Characteristics of the intervention (e.g. relative advantage, complexity, observability and compatibility)
	Development process	Adopters and implementers perceptions toward the development process of ISAC (e.g., credibility of developers, objectivity and transparency)
	Organizational characteristics	Characteristics related to implementing organization; Jazan Dental School (JDS) (e.g., implementation environment, guidelines, clinical time) as well as the outside JDs environment
	Socio-political context	Characteristics of the social and political context in which ISAC intervention will be implemented (e.g., dentist role in society, conflicting barrier)
	Motivations for use	Factors that can increase dental interns, adopters or implementer motivation to use/adhere to ISAC

**Table 3:** Summary of themes, sub-themes and illustrative quotes

1-What are the first thoughts, feelings or impressions of dental interns while practicing ISAC?			
**Fleuren’s framework**	**Theme**	**Sub-theme**	**Example Quote**
User level of determinants	Immediate feel (Affective attitude)	Regret	*“I am regretting that I was not fair with patients”-DentalIntern4*
		Professional	*“…it gives me the feeling that the doctor is professional and respectful.”-DentalIntern4*
		Organized	*“I feel more organized”-DentalIntern6*
		Confidence	*“It builds my confidence in myself.”-DentalIntern2*
		Awareness	*“I feel I am highly aware of oral cancer screening more than before”-DentalIntern3*
	Recommendations	ISAC clinical protocol	*“We need ISAC clinical protocol to ease following the steps”-DentalIntern3*
		Use the Arabic language	*“It would be easier for us if the materials were in the Arabic Language”-DentalIntern1*
		Divide training per each intervention component	*“It might be good if I have separate training on tobacco counseling and patient communication before actual implementation of ISAC.” – DentalIntern 6*
2-What are the adopters, implementers and dental interns’ perceptions toward ISAC intervention?			
**Fleuren’s framework**	**Theme**	**Sub-theme**	**Example Quote**
User level of determinants	knowledge	Aim of intervention	*“…it is a comprehensive program aims to increase students’ awareness themselves aaa and the comprehensive goal is the early detection of oral cancer” – Adopter1*
		Model of intervention	*“It rests on sound evidence-based methods, and it covers most of the deficient parts observed in dentist practice for example tobacco training and soft communication skills with patients.” – Adopter2*
	Cognitive attitude	Serves country needs	*“…it targets the country need.” – Implementer2*
		Right project in right time	*“It is the right project in the right time. We really need for such intervention”-Adopter1*
	Change beliefs	High ability to change	*“…ISAC is going to help us from the perspective of early detection and from the perspective of the skills and abilities gained from ISAC” – Implementer1*
	Barriers	Gender	*“…I don’t think all females would be satisfied that males examine them especially in the neck region and behind the neck.” – Adopter1*
		Language	*“The second point that could be a problem is the language barrier and its less in Jazan region because most of patients are Arabic speakers and therefore patients can understand them, and they can understand patients.”-Adopter2*
		Dentist not from the region	*“A point that could be present before, that the dentists were not from the same region, and they probably may not know the region needs and problems”-Adopter2*
	Facilitators	Gender concordance	*“Among the points that should be highlighted is that usually dentists available in both gender and patients usually distributed based on gender. Most male patients go to male doctors and* vice versa*.”-Adopter2*
		Supportive JDS^a^ environment	*“The JDS environment is very supportive for such an intervention.”-Adopter2*
		Regional trainers	*“I wish that you bring doctors who practice in the region, and they know how to deal with patients and problems in this region”-DentalIntern1*
		Standard form	*“What I really like in the ISAC is that it tries to bring new form that we can easily follow it and if possible, I wish that you share it with ministry of health”-DentalIntern*
		Collaboration with Ministry of Health	*“I suggest that after we finished the project that the ISAC team prepare special presentation for the program to the ministry of health so they will be in the same image.”-DentalIntern2*
	Motivation to use	Appealing	*“The program is already appealing. We learned effective communication and smoking cessation counseling”-DentalIntern1*
		Certificates	*“If dental interns will have certificates, they will be more excited for the program.”-Implementer2*
		Easy and clear	*“Because it is easy to learn and to practice”-DentalIntern4*
		Build confidence	*“One of the things that attracted me is that it has built my confidence on my ability to do the screening.”- DentalIntern2*
		Role model	*“The fact that Dr. Jafer is the provider makes me excited and motivated for practicing ISAC.”-DentalIntern3*
		Impact of oral cancer	*“I think the important thing is that they know the impact of oral cancer.”- Adopter1*
		Advertisement	*“To be appealing, I think the university should make it visible to students especially the students and social media.”-DentalIntern1*
Innovation level of determinants	ISAC development	Credible developers	*“I believe that the developers are among the most qualified in the college and they know the region needs. They have diverse experiences; they have administration experiences, academic experiences, and clinical experiences, has work in academia and in ministry of health. They have good networking and experience in research and publications, and they are specialized in dental public health and exactly in disease prevention and creating intervention”-Adopter2*
		Engagement	*“It rests on studies and involved students and different groups including us.”-Implementer1*
		Scientific approach	*“I think the creation of the intervention follows clear scientific steps. Therefore, I believe this project was born to be successful.”-Adopter2*
		Clear process	*“Everything was really clear… I felt I was guided step by step from the beginning”-Adopter1*
	Relative advantage	Targets dentists	*“What I like in your intervention is that it targets oral healthcare providers themselves.”- Adopter1*
		Comprehensive	*“Previous program didn’t ask if you use Shammah or not. I mean the education was superficial not in-depth like in the ISAC”- Implementer1*
		Raise the attention to oral cancer	*“But we know that these conferences were the result of the ISAC founders’ efforts to the problem.”- Adopter2*
		Well-defined objectives	*“I am not aware of any program that was like this or that targets oral cancer. This program is based on clear objectives and thorough assessments.”- Implementer2*
		Use relevant evidence	*“The most thing that I like, and I feel it’s a new thing is that it is built on information that is relevant to our field and our community.”-DentalIntern1*
	Complexity	Different forms	“We don’t have a standard way that everyone can use in all the provinces of Jazan region.”- DentalIntern6
		Easy to practice	*“It’s easy to learn and practice”-DentalIntern2*
		Clinical time outside JDS	*“We have limited time. We are practicing outside the college. So, we need to train on it many times to fit the clinical time.”- DentalIntern5*
		More training	*“…we need more time for it. For example, patient education and communication, we need to practice them more”-DentalIntern5*
	Observability	Depends on measures	“It depends on the way of your measurements.”- Adopter1.
		Very visible	*“The result will be visible for everyone... I feel it will make a big change.”- Implementer1*
	Compatibility	Highly compatible with society norms and beliefs	*“I see that it is 100% compatible because it arises from the community needs.” – Adopter2*
Organizational level of determinants	Implementation environment	Supportive in JDS	*“But outside the college, the supervising doctor tells me to check directly the patient chief complaint. So, I think I will practice ISAC if I have space for it. But if the doctor forces me, I don’t know what to do?”- DentalIntern4*
	Guidelines	Consistent with JDS guidelines	*“…the college mission includes healthcare delivery, and this project optimizes the healthcare. So, I expect that it will support it to a large extent.” – Implementer2*
	Clinical time	Fit within existing time	“I think that because we are not organized in screening and in the steps that we should do it will take time. But I believe I can screen within the same clinical time that I usually have. I saw when the doctor performed it he was very organized and did not take long time”-DentalIntern1
Socio-political context	Dentist’s role in society	Important	*“I see their role is important…Only the dentist knows the mouth very well. The advice that the dentist gives can increase patient knowledge and awareness about oral cancer. The dentist can screen very well for oral cancer and teach the patients on screening.”- Implementer1*

^a^*JDS* Jazan Dental School

## User level of determinants

### Knowledge

Participants have accurately captured the model and the aim of the ISAC intervention as follows: a comprehensive and evidence-based intervention that fills a gap in dentist practice toward oral cancer and aims to enhance oral cancer early detection.

### Cognitive and affective attitudinal beliefs

Participants perceived the ISAC as an excellent, successful intervention and the right project at the right time. Further, they perceived the ISAC as an intervention that targets the needs in the country. Regret of having been unfair to patients before, high confidence and awareness, more organized patient examination and professionalism were the feelings reported by participating dental interns.

### Change beliefs

Participants anticipated that the ISAC will create positive changes in terms of dentists’ skills, confidence and early detection of OC.

*“…the ISAC is going to help us from the perspective of early detection and from the perspective of the skills and abilities gained from ISAC”* – Implementer1

### Potential barriers/facilitators

Two participants believed that gender of both patient and dentist can be a possible barrier to practice the ISAC intervention outside JDS.

*“…I don’t think all females would be satisfied that males examine them especially in the neck region and behind the neck.”* – Adopter1

Other participants believed that it might appear as a barrier in research more than in the actual dental practice in the region. Also, they believed that with increasing dentists and patients’ awareness as well as dentist-patient communication skills, gender would no longer be a potential barrier of practicing ISAC.

Participants mentioned some potential facilitators for the ISAC implementation in JDS including gender-concordance, supportive environment, use of regional trainers, standard examination forms as well as collaboration with the Ministry of Health (MOH).

### Motivation to use

Participating adopters believed that increasing dentist’s awareness about the impact of OC on patients will motivate them to practice the ISAC. Further, participating implementers believed that giving dental interns certificates for performing the ISAC can motivate dental interns to practice the ISAC. Some of participating dental interns have shared a similar point but mentioned that having a certificate for performing the ISAC is not the main reason that would motivate them. Many participants found that the ISAC intervention is already appealing for them and few participants mentioned the fact that the ISAC has built their confidence is one of the things that made the ISAC attractive to them. Others mentioned that feeling satisfaction in following religious teaching is what motivates them to practice ISAC. Some participating dental interns believed that making advertisement for the ISAC can increase the attraction to the intervention. Many participants mentioned that the program provider himself was a motivator for them to practice the ISAC.

## Innovation level of determinants

### ISAC development

All participants shared favorable perceptions toward the transparency/clarity of the ISAC development and developer credibility. Among the points that were mentioned in relation to objectivity and clarity was the use of a scientific approach as well as the engagement of different groups in the ISAC development and planning process such as students and dental interns.

### Relative advantage

Participants perceived that the ISAC has a superior advantage in comparison to the previous program, which is the fact that ISAC is targeting oral healthcare providers. Whereas previous programs targeted people in the community with unknown characteristics. Participants mentioned that there was no previous program that included an intervention like the ISAC and those previous implemented programs were focused only on raising awareness about the negative health effects of using Shammah. Two participants believed that the ISAC developers have raised the attention to OC in the Jazan region because of the ISAC development process and subsequently the organization of two international and national conferences.

*“But we know that these conferences were the result of the ISAC founders’ efforts to the problem.”-* Adopter2

Majority of participants believed that the ISAC has well-defined objectives that makes it superior to previous programs related to OC. Dental interns favored that ISAC was built on relevant information and data in relation to the dental field and Jazan culture. For example, including dental interns like them in the first stages of the needs assessments and using their output in the development of the ISAC.

### Complexity

Majority of participants believed that the ISAC is easy to be practiced. Some participants mentioned that the ISAC is not difficult but requires training. Others mentioned that they may suffer difficulty in practicing the ISAC outside JDS as they may have very limited clinical time in a future dental daily practice as compared to the situation in JDS context. Two participants stated that the variation in the examination forms between JDS and other clinics might make it difficult to practice the intervention.

### Observability

Majority of participants believed that the results from the ISAC intervention will be very visible to others. Others believed that the visibility of the results will depend on the effect measurement.

### Compatibility

All participants found the ISAC intervention very compatible with the norms and beliefs in the society.

*“I see that it is 100% compatible because it arises from the community needs.”* – Adopter2

## Organizational level of determinants

Participants perceived the JDS environment as supporting for the intervention. Some participants mentioned that the clinical environment outside JDS may not be supportive of OC detection as included in the ISAC intervention as they have limited time and their supervising doctors may focus only on patient’s chief dental complaint. In relation to the ISAC intervention fitting to organizational guidelines, participating dental interns were not aware about the organization guidelines. The participating adopters and implementers believed that the ISAC intervention is consistent with the JDS organization guidelines.

*“…the college mission includes healthcare delivery, and this project optimizes the healthcare. So, I expect that it will support it to a large extent.”* – Implementer2

## Sociopolitical context

Participants mentioned that currently dentists are not screening for OC and not educating patients. They believed that dentists are in the front line against OC and that they play an important role in the society as they are the most qualified professionals to detect OC at an early stage and therefore, they should screen for OC and educate their patients about it.

### Recommendations by dental interns

Participating dental interns stated that they could easily memorize the main four steps of the ISAC in general. But they struggle to remember all the sub-behaviors related to each step. They have stated it would be very helpful if they had the ISAC clinical chart/protocol with the steps they need to follow. Further, they felt they need more practice on patient education and preferred to receive separate training on patient communication and tobacco counseling before the ISAC comprehensive training. In addition, they preferred to have the ISAC materials given in Arabic language.

## Discussion

Overall, participants shared favorable beliefs and expectations about the ISAC intervention and its ability to increase the early detection and prevention of OC. Including representers from different relevant groups related to the OC problem in the conceptualization and the development of the intervention was highly valued by the participants. In fact, engaging stakeholders and those who might be affected by the intervention was found to be associated with high trustworthiness and perceived relevance to these groups [14, 23]. Health interventions are more acceptable to the community and the target participants when the research that has generated the evidence-based intervention does not originate from different circumstances and places [24]. In the early stage of the intervention development (in-depth needs assessment), we found that dental interns had low confidence and lack the necessary skills of oral cancer examination and patient education. Therefore, the ISAC intervention was developed to effectively enhance their confidence and skills toward oral cancer examination and patient education. The engagement of dental interns, clinical director, interns’ supervisor, and community dentistry specialists from the early stages of the intervention development facilitated designing and developing intervention materials and instruments that are relevant and sensitive to dentists’ needs, better tailored, more likely to be implemented and eventually would be more effective [14]. Among the organizational level factors influencing the implementation of a healthcare intervention based on the Fleuren framework, is the available time and organizational support [15]. These factors are of great importance to the success of the ISAC implementation and they differ from organization to organization. In the context of JDS, participants perceived the faculty leaders as positive, supportive and that the available clinical time is sufficient for the practice of ISAC. Participating dental interns expected that practicing in other dental organizations might be challenging in terms of available time and organization leaders’ attitudes and their focus on patient’s chief dental complaint. Thus, the observed behavioral change may not be sustainable, and dentists may struggle in adhering to the ISAC practice. This challenge is expected to be diminished when the ISAC intervention is adopted by the other organizations as we planned in the following steps.

Having short-term visible changes can enhance the adherence [7, 25]. Which is in line with our findings, participants expressed that feeling confident makes them excited to continue practicing the ISAC. Other visible changes experienced by the participants included feelings of being professional and organized. Others felt regret of not being fair to previous patients. Which might be viewed as a form of self-reevaluation that is a critical motivator for individual’s change [26].

A majority of participants mentioned that presence of certain academic staff as the ISAC provider was a motivator for the ISAC practice. The motivational theory of role modeling had revealed the great influence of the role models and that it can be used to rise role aspirants’ motivations and reinforce their goals [27]. Satisfaction in following the Islamic teaching that encourages saving others’ lives and altruism are perceived by some participants as their motivator. In contrast to the certificates and role model, this type of motivation can be viewed as more intrinsic, autonomic and associated with self-determined behavior that is more likely to be maintained [28].

A particular strength of this study was the use of the Fleuren framework which helped us to understand the complex context in which the ISAC would be implemented. Another strength for the study is the utilization of member checks during and after the interviews. We have utilized member checks to enhance the validity, credibility and transferability of the findings in a qualitative study [29]. To facilitate transferability judgment by other researchers (external validity in quantitative research), we’ve provided thick and rich descriptions of the participants’ perceptions as well as the study context [30].

There were some limitations of this study which represent opportunities for future research on adoption and implementation of an intervention in a dental healthcare setting. The data reflected in this study was recruited from a local context, therefore the findings of this study should be interpreted with precaution when transferred to another setting.

### Implications for practice

This study was done on the major dental organization in the Jazan region; thus, it would be interesting to explore other dental organizations perceptions, opinions and possible challenges toward the implementation of the ISAC intervention in their daily practice. In line with other studies, the study findings support the idea that following a co-participatory approach throughout the project, enhances intervention compatibility, perceived relevance and engagement of the target group. This study included dental interns’ perceptions of potentially embedding factors for implementing and practicing a comprehensive OC examination in dental organizations other than JDS. The significant feedback from the participating dental interns should give current dental interns practicing outside JDS confidence to negotiate and push their organization leaders’ focus of attention from focusing only on patient complaint to include a serious community issue as OC and to facilitate the use of an evidence-based intervention that tackles the disease. Dental interns and other oral health providers should advocate for the active practice of OC examination and consider the ramification of dentists’ passive behavior on patient quality of life and OC burden on the community. Leaders and directors of dental organizations should take their responsibility toward the OC issue, facilitate and embolden OC examination in their clinical practice. Finally, participants feeling of immediate intervention changes on their confidence and skills has motivated them to continue practicing ISAC in their daily clinical practice. Intervention developers should pay attention to combining long-term change with short-term visible changes that can enhance participants’ adherence to the intervention. The ISAC intervention was meant to eliminate the observed barriers and challenges in dental interns’ awareness, confidence, and skills toward the practice of OC examination and patient education. Dental internship is the first stage when graduates of dental school independently practice in the clinics (with zero to minimal interfere from senior dentists) and they are considered practicing dentists. Practicing dentists with considerable years of experience can benefit from practicing the intervention after receiving the necessary education and training on the ISAC. To apply the ISAC for instance on a national level of Saudi Arabia, where all dentists would be practicing the intervention, we may need further exploration of the needs, facilitators, and barriers perceived by practicing dentists in relation to further implementation of the ISAC.

## Conclusions

Integrating data from representers of different participant groups during intervention conceptualization and development are critical for intervention compatibility and acceptability by the community. The study findings reflected on the opportunities of intertwining intrinsic motivators existed in the target group as satisfaction and altruism with extrinsic motivators as certification to boost and sustain the behavior change. Further, visible short-term changes and presence of inspirational role models enhanced participants’ motivation to practice the intervention.

## Supplementary Information

**Additional file 1.** Semi-structured interview protocol.

**Additional file 2.** Coding tree.

## Data Availability

The datasets used and analyzed during the current study are available from the corresponding author on reasonable request.

## Abbreviations

OC: Oral cancer
CDD: Community dentistry division
ISAC: I = inform, S = screen, A = advice and C = connect
IMA: Intervention mapping approach
JDS: Jazan dental school
MOH: Ministry of Health
