# Supplementary material for: Dentists’ perceptions and usability testing toward the implementation of the ISAC, a comprehensive oral cancer intervention in dental practices: a qualitative study in Jazan region, Saudi Arabia
Source: BMC Health Serv Res. 2022 Feb 12;22:187. doi: 10.1186/s12913-022-07586-2 (PMC8840285; doi:10.1186/s12913-022-07586-2)
Supplement: Supplementary file 1 — Additional file 1. Semi-structured interview protocol. [file 12913_2022_7586_MOESM1_ESM.docx]

**Focus Group Discussion Guide for Dental Interns**

A) What do you think toward ISAC:

1. Relative advantage: degree to which ISAC is perceived as better than the idea it supersedes
2. Complexity: degree to which ISAC is perceived as relatively difficult to understand and practice
3. Compatibility: degree to which ISAC is perceived as consistent with existing beliefs and values, past experiences, and needs
4. How do you perceive the support of JDS toward oral cancer practice and toward such intervention?
5. How does ISAC intervention fit within JDS guidelines?
6. What do you think about the time it takes to perform ISAC activities along your other activities as a dentist?
7. How do you perceive the role of dentists in oral cancer prevention and early detection in Jazan?
8. If any, what are the possible barriers that may conflict with ISAC implementation?
9. What would make ISAC more appealing to you?
10. What would make you adhere to using ISAC?
11. Is there any other comment that you would like to add?

Thank you for your participation.

**Thinking aloud:**

Please express what you feel or what you think during practicing ISAC.

What do you think about the ISAC presentation and materials?

**Semi-structure interview guide for Adopters**

Thank you for accepting the interview invitation about ISAC project although you have a busy schedule. How are you? When you are ready, I would like to take few minutes of your time to ask you some questions about ISAC intervention.

1. What do you know about ISAC intervention?
2. What do you think of ISAC?
3. In general, what do think or how do you feel about the change?
4. What do you think of ISAC ability for creating change?
5. As adopter, what concerns do you have about ISAC adoption/implementation?
6. What do you think is the solution for such issue?
7. How do you see/ what do you think of the development process of ISAC?
8. To which degree do you perceive ISAC is better than the other superseding intervention/programs?
9. To what degree do you perceive ISAC is relatively difficult to understand or practice?
10. To which degree do the ISAC results can be visible?
11. To which degree do you think ISAC intervention is compatible with the norms, needs and values?
12. Is there anything you would like to add before ending the interview?

Thank you very much for your time and your feedback

**Semi-structure interview guide for Implementers**

1. As implementer or responsible authority to implement and train interns or students on ISAC, is what do you know or what did you know about ISAC intervention?
2. What do you think about it?
3. What do you think of ISAC ability for change?
4. what concerns do you have about ISAC implementation?
5. What would be the things that facilitate for us the implementation of ISAC?
6. What do you think about the credibility of developers?
7. To which degree do you perceive ISAC is better than the other superseding intervention/programs?
8. What about its difficulty? Is implementing ISAC challenging?
9. What about its compatibility with the norms, ethics, needs and values?
10. What about its trialability of ISAC?
11. How can we ensure interns high adherence to ISAC practice?
12. How do you perceive the support of JDS regarding the intervention?
13. How do you perceive the role of dentists in oral cancer prevention and early detection in Jazan?
14. What could conflict with implementing ISAC?
15. What other dentists might think of this intervention?
16. Any point you would like to add or any question you have?

Thank you very much for your time and your feedback
