# Supplementary material for: Dentists’ perceptions and usability testing toward the implementation of the ISAC, a comprehensive oral cancer intervention in dental practices: a qualitative study in Jazan region, Saudi Arabia
Source: BMC Health Serv Res. 2022 Feb 12;22:187. doi: 10.1186/s12913-022-07586-2 (PMC8840285; doi:10.1186/s12913-022-07586-2)
Supplement: Supplementary file 2 — Additional file 2. Coding tree. [file 12913_2022_7586_MOESM2_ESM.pdf]

Coding Tree Map: Main themes and sub-themes identified from the qualitative analysis using NVivo software for the interview and focus group discussion.

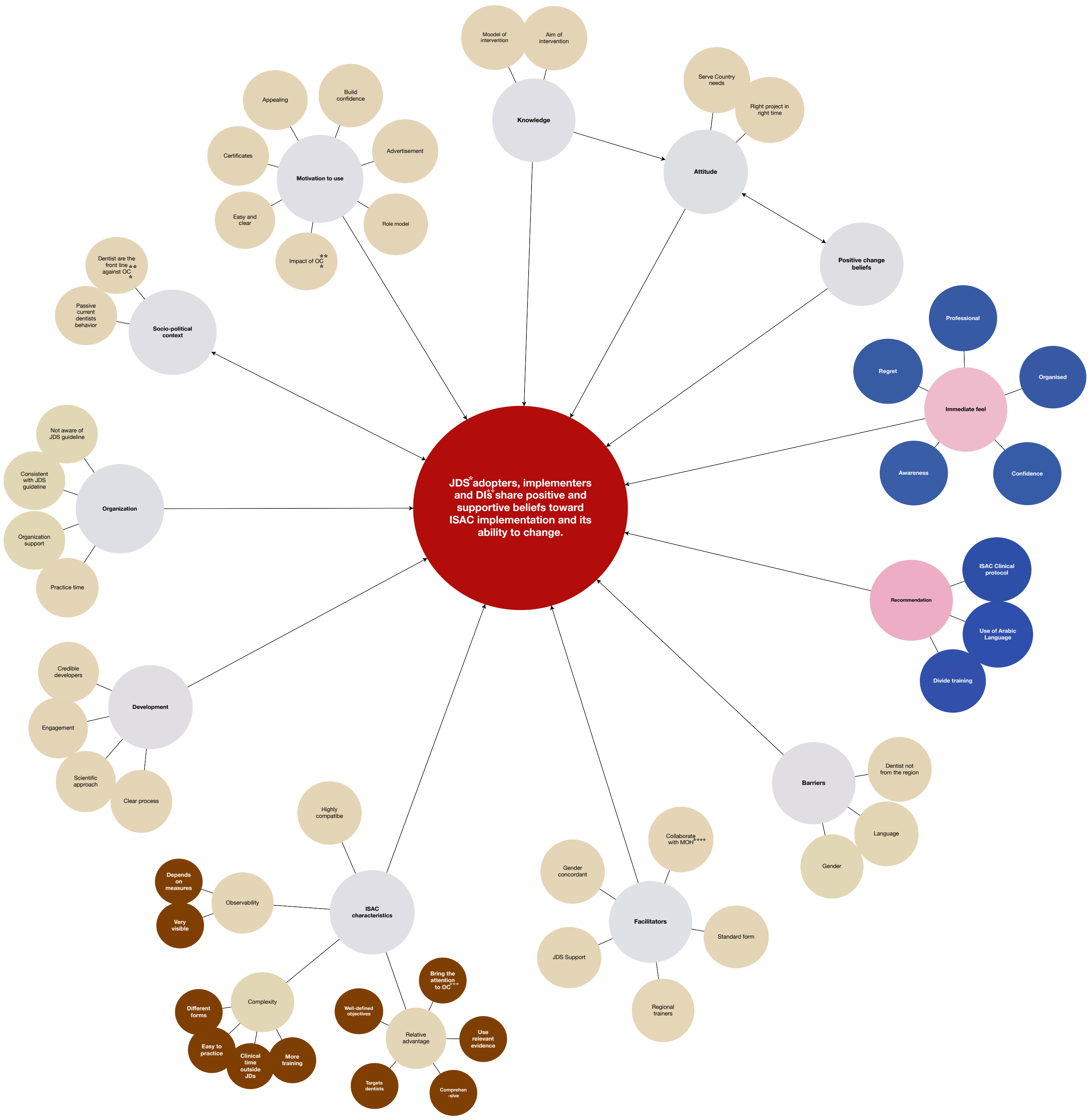

*JDS: Jazan Dental School; **DIs: Dental Interns; ***OC: Oral Cancer; ****MOH: Ministry of Health
